# Japanese genomes for pharmacogenomics: primary and secondary pipelines for population-specific insights

**DOI:** 10.3389/fbinf.2026.1770550

**Published:** 2026-03-19

**Authors:** Charles W. Crawford, Yuka Nakano, Marika Hayashi, Antony Ibrahim, Issei Kono, Iri Sato-Baran, Takeshi Ozeki

**Affiliations:** Genesis Institute of Genetic Research, A.D.A.M. Innovations Corporation, Shibuya-ku, Japan

**Keywords:** Japan, opioids, PGx, pharmacogenomics, pipelines, SSRI, statins, whole genome sequencing

## Abstract

Pharmacogenomics (PGx) incorporates population level allele frequencies into its analyses. Often these population groupings are based on current or ancestral geographic location. However, these groupings can obscure internal variance caused by population heterogeneity. In order to increase the data accuracy and specificity for researchers, it is necessary to refine the population groupings. Often the necessary datasets have already been collected but have not been fully analyzed past their initial purpose. Here we provide a secondary pipeline that demonstrates a divergence between two datasets: the Clinical Pharmacogenetics Implementation Consortium (CPIC) collection of geographic populations and a Whole Genome Sequencing PGx dataset consisting of 632 Japanese individuals. Three classes of drugs and the relevant genes as identified by CPIC are examined: SSRIs (CYP2D6, CYP2B6, CYP2C19), opioid analgesics (CYP2D6) and statins (SLCO1B1). A meaningful divergence is shown between CPIC’s East Asian population and the Japanese population for opioid analgesics and statins. For opioid analgesics the Japanese population saw an increase in the “Use as directed” designation compared to the East Asian population from 53.2% to 71.0%; the statins data showed a decrease from 75.7% in the East Asian population 67.6% in the Japanese population. This divergence demonstrates that existing WGS data can reveal PGx patterns masked by broad geographic groups through the application of an appropriate secondary pipeline, enabling 0population specific implementation and refined population-level PGx inference without the need for further sample collection.

## 1 Introduction

Population groupings are commonly used in pharmacogenomics to represent the variation between demographic groups and could be of clinical utility (Lakiotaki et al., 2017). For example, the genetic polymorphisms that result in increased CYP2D6 metabolism are primarily observed in Africans and were linked to negative outcomes for tramadol and codeine (Zhou et al., 2017). However, these groupings, by their nature, can obscure variations in subpopulations (Bailliet et al., 2007; Patrinos et al., 2023). For example, indigenous groups in Brazil have been found to vary in HLA-A*31:01 frequency, while still living in the same indigenous reservation area (Fernandes et al., 2022). Japan presents a useful example: while it is often grouped under the broad category of “East Asian” in global Pharmacogenomics (PGx) resources, centuries of demographic isolation and bottlenecks have left the Japanese population with distinctive allele frequencies that could meaningfully affect drug response (Kisoi et al., 2020; Kawai et al., 2023). Accurate PGx data requires the proper grouping of individuals into genetically meaningful populations and not relying on convenience samples or geographic proximity alone. Resolving the ambiguity of the population groupings is now feasible as the past decade has produced an increase in Whole Genome Sequencing (WGS) projects, both in research or academic contexts and through direct-to-consumer genomics services with the increase in available data (Yang et al., 2025; Samlali et al., 2022).

The increase in WGS data acquired for other research programs can be repurposed for population level PGx analysis, as we will show here. The processing of genomic data is broken down into primary, secondary and tertiary analysis. The output of the final stage of analysis results in annotated variants that then can be filtered based on the parameters of the study (Austin-Tse et al., 2022) and then further processed into the relevant units of analysis. However, different paths from the annotation step or later could be used to deliver useful data that is orthogonal to the dataset’s primary purpose. For example, the data could be filtered on PGx relevance after the primary use case has been accomplished. This additional processing we will refer to as the secondary pipeline. The primary pipeline being the final step in tertiary analysis that provides the necessary final data product for which the genetic samples where acquired. In our case, a secondary pipeline focused on population level analysis has been used to extract further data after a primary pipeline focused on individual measurements has been completed.

Here we demonstrate that, from an existing WGS dataset (collected by A. D.A.M. Innovations Co. (Japanese Corporate name: Genesis Healthcare Co.)), clinically relevant population-level estimates can be derived and used to uncover divergences between aggregated populations within the publicly available Clinical Pharmacogenetics Implementation Consortium (CPIC) data (Gene Reference Materials for CYP2D6, 2022; Gene Reference Materials for CYP2B6, 2025; Gene Reference Materials for CYP2C19, 2020; Gene Reference Materials for SLCO1B1, 2024). We used this output of a PGx pipeline focused on collating individual results to construct a secondary pipeline focusing on population level insights for a Japanese-focused dataset. This secondary pipeline and the population specific privacy-conscious, secondary PGx database are both outlined. The results enable new comparisons to global resources such as the CPIC, highlight both alignment and divergence between Japanese and broader East Asian data and broaden the value of the initial research, raising possibilities for future research and a better understanding of the diversity of pharmacogenomically-relevant genes.

## 2 Methods

### 2.1 Dataset construction

**FIGURE 1:**
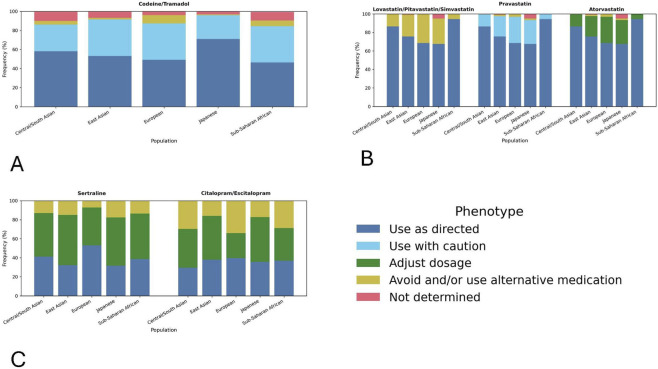
PGx recommendation frequencies for 3 drug categories by population **(A)** Opioids (Codeine, Tramadol) **(B)** Statins and **(C)** SSRIs.

The initial dataset consisted of 632 (631 for citalopram and escitalopram) participants who underwent clinical-grade WGS through A. D.A.M. Innovations’ consumer testing service. A central feature of the secondary pipeline was its explicit focus on Japanese ancestry. All 632 participants were selected from a larger undifferentiated dataset, as shown in Figure 1. To generate the robust, representative Japanese dataset, a threshold of ≥87.5% Japanese ancestry was adopted, done with FastNGSadmix in comparison (Jørsboe et al., 2017) to the 1000 Genomes Project’s Japanese population panel (Japanese in TokyoJapan, 2025). ≥87.5% was selected to match the 1,000 Genome Project’s requirements of 4 grandparents born in Japan.

### 2.2 Sample & data processing

Saliva samples were collected, DNA was extracted, and sequencing performed via whole-genome shotgun resequencing. Briefly, DNA was extracted from saliva and purified with Oragene prepIT (DNA Genotek, Ottawa, Canada). Libraries were prepared using the MGIEasy FS DNA Library Prep Set (MGI Tech Co., Ltd., Guangdong, China). WGS analysis was performed using a DNBSEQ-T7 (MGI Tech Co., Ltd., Guangdong, China) for sequencing and the Illumina DRAGEN DNA pipeline Ver. 3.9.3, Illumina, San Diego, CA) for read mapping using hg38 reference and with following options: remove-duplicates = false, enable-cyp2d6 = true. enable-variant-caller true, vc-emit-ref-confidence = BP_RESOLUTION. The QC thresholds are as follows: Average sequenced coverage over genome ≥20–30×, call rate of target bed ≥90%.for read mapping. From these variant calls, three tools were used to generate pharmacogenomic interpretations. First, the Illumina DRAGEN CYP2D6 caller, incremented in the Illumina DRAGEN DNA pipeline (Ver. 3.9.3, Illumina, San Diego, CA), was used for genotyping CYP2D6, a gene with complex structural variation and copy number variation that requires specialized algorithms (Chen et al., 2021). Second, T1K (Ver. 1.1.7-r225, The ONE Genotyper for KIR and HLA) was used for high-resolution genotyping of HLA-A and HLA-B (Song et al., 2023), both of which are clinically relevant for adverse drug reactions such as carbamazepine-induced Stevens–Johnson syndrome (Subramanian et al., 2025). Finally, PharmCAT (ver. 2.15.3) was used for calling and interpreting remaining PGx genes, mapping alleles to star-allele haplotypes, phenotypes, and associated CPIC guidelines (Sangkuhl et al., 2020).

The outputs of these three tools were harmonized into a common format, where drug recommendations mapped to one of six categories: “Use as directed,” “Adjust dosage,” “Avoid and/or use alternative medication,” “Use with caution,” “Not determined,” or “No call.” CPIC provides clear mappings from diplotypes to phenotypes and from phenotypes to a recommendation. Sertraline shows this well as the recommendation is dependent on two genes CYP2B6 and CYP2C19 (Gene Reference Materials for CYP2B6, 2025; Gene Reference Materials for CYP2C19, 2020). Referring to CPICs Diplotype/Phenotype tables we can classify each individual as a Rapid, Normal, Intermediate or Indeterminate metabolizer of Sertraline with respect to the CYP2B6 and CYP2C19 genes. With these two phenotypes we can determine the recommendation for the individual by referencing CPIC’s publications, in this case Table 5 of “Clinical Pharmacogenetics Implementation Consortium (CPIC) Guideline for CYP2D6, CYP2C19, CYP2B6, SLC6A4, and HTR2A Genotypes and Serotonin Reuptake Inhibitor Antidepressants” [add citation]. This can be done for any data set that provides the diplotype information for the population. With these classifications and the ancestry filter used, a dataset that can be easily compared to the CPIC results was generated, allowing for direct population comparison. It is noted that the definition of “Not determined” includes “Unknown function” (no literature describing function, or the allele is novel) and “Uncertain function” (literature supporting function is insufficient, conflicting or weak) for pharmacogenes except for HLA-A and HLA-B. In contrast, for HLA-A and HLA-B, the criteria for determining the function of the allele are only “positive” (detection of high-risk allele) or “negative” (high risk-allele not detected); therefore, “Not determined” does not exist.

### 2.3 PGx database construction

In the primary pipeline, to balance clinical utility with data protection, a strict separation of identifiers was implemented. Each customer purchases a kit and all results are tracked through the kit. Each customer has a user id and each user id is associated with any purchased kits through kit ids. PGx results were linked only to kit ids, not directly to user ids. Demographic information (age, sex, purchase location, et c.) is stored in a separate database and merged only at the secondary analysis stage. This secondary database aggregates only the necessary data allows population-level insights without exposing individual-level identifiers, while still allowing filtering by age, sex and purchase location. The final database only includes age, sex and purchase location and a kit id or generic id for internal or external use respectively. The database schema can be found in Supplementary Figure S1. To optimize performance for analysis, data was aggregated from essential fields into a single SQLite database.

### 2.4 Data sharing

The database was made internally accessible within A. D.A.M. Innovations through an Amazon Web Services (AWS) hosted instance of Metabase (Metabase, Spring Valley, CA), an open-source platform for data visualization and dashboarding. An example screen can be found in Supplementary Figure S2. This provides clinicians, bioinformaticians, and non-specialists with access to PGx data, supporting both hypothesis generation and operational decision-making. In the Supplementary Material are the schema and frequency tables for all the drugs reviewed in this paper.

## 3 Results

Our goal is to provide a secondary pipeline that compares a Japanese population data set to CPIC’s data by filtering for genetic ancestry, linking diplotype to phenotype, selecting drug categories for comparison, comparing different populations and, where possible, corroborating with third party data. Starting with our database of individual PGx results, we filtered by Japanese genetic ancestry and then processed the resulting data into an anonymized database focused on demographic analysis. As a proof of concept, we compared A. D.A.M. Innovations’ Japanese PGx dataset against CPIC population reference data across three drug categories: Selective serotonin reuptake inhibitors (SSRIs) (citalopram, escitalopram, and sertraline), Statins (atorvastatin, pravastatin, lovastatin, pitavastatin and simvastatin), and Opioid analgesics (codeine and tramadol). Four genes covered these therapeutic categories: CYP2D6, CYP2B6, CYP2C19, SLCO1B1, (Gene Reference Materials for CYP2D6, 2022; Gene Reference Materials for CYP2B6, 2025; Gene Reference Materials for CYP2C19, 2020; Gene Reference Materials for SLCO1B1, 2024). For each of the drugs, the recommended use frequencies were extracted from 4 CPIC populations plus the A. D.A.M. Innovations data. Where possible, corroborating data was found for any discrepancies between the East Asian and Japanese populations.

These three drug categories were chosen because they are widely prescribed, have clear PGx guidelines, and illustrate differences in allele frequencies that may alter prescription choices (Zhao et al., 2025; Lalji et al., 2021). For SSRIs, CYP2C19 metabolizer status is a key determinant of dosing and drug choice (Bousman et al., 2023). In CPIC data, East Asian populations exhibit higher rates of poor metabolizers compared to Europeans. For sertraline, CYP2B6 variation adds further complexity, again highlighting the need for population-specific data (Bousman et al., 2023). For statins, variants in SLCO1B1 are associated with risk of statin-induced myopathy (Cooper-DeHoff et al., 2022). For opioids, CYP2D6 ultrarapid metabolizers and poor metabolizers present risks for codeine and tramadol efficacy and toxicity (Crews et al., 2021). By leveraging DRAGEN’s specialized CYP2D6 caller, we generate high-confidence genotype calls, revealing Japanese-specific distributions of activity scores that could inform local prescribing policies after validation.

Figure 1 shows the frequency of PGx designations for 5 populations. Four are from the CPIC datasets: Central/South Asian, East Asian, European and sub-Saharan African. The remaining dataset is the Japanese population from the A. D.A.M. Innovations dataset. From Figure 1, variations in the populations selected by CPIC can be seen. For example, there is a clear distinction between the Sub-Saharan population and the other populations in the SSRI dataset. This supports the utility of these groupings, but our dataset shows some limitations.

For finer-grained comparison, Table 1 reports the East Asian and Japanese (along with an additional dataset for opioids) trait frequencies together with 95% confidence intervals to assess whether the population estimates differ. Confidence intervals were calculated using the Wilson score method, which provides more reliable coverage than the standard Wald approximation, particularly for proportions near 0 or 1 (Newcombe, 1998). Supplementary Table S1 includes the full table of frequencies for all population groupings.

**TABLE 1:** Table of comparing the East Asian and Japanese PGx recommendation Frequency data.

Category	Drug	Population	Phenotype	N (population)	Frequency	95% CI (wilson)
Statins	Lovastatin/pitavastatin/simvastatin	East Asian	Avoid and/or use alternative medication	2,471	23.5%	21.9%–25.2%
			Not determined		0.8%	0.5%–1.2%
			Use as directed		75.7%	74.0%–77.3%
		Japanese	Avoid and/or use alternative medication	632	27.5%	24.9%–31.9%
			Not determined		4.9%	3.1%–6.3%
			Use as directed		67.6%	63.6%–70.9%
	Pravastatin	East Asian	Avoid and/or use alternative medication	2,471	1.6%	1.2%–2.1%
			Not determined		0.8%	0.5%–1.2%
			Use as directed		75.7%	74.0%–77.3%
			Use with caution		22.0%	20.4%–23.6%
		Japanese	Avoid and/or use alternative medication	632	1.7%	0.9%–3.0%
			Not determined		4.9%	3.1%–6.3%
			Use as directed		67.6%	63.6%–70.9%
			Use with caution		25.8%	23.3%–30.2%
	Atorvastatin	East Asian	Adjust dosage	2,471	22.0%	20.4%–23.6%
			Avoid and/or use alternative medication		1.6%	1.2%–2.1%
			Not determined		0.8%	0.5%–1.2%
			Use as directed		75.7%	74.0%–77.3%
		Japanese	Adjust dosage	632	25.8%	23.3%–30.2%
			Avoid and/or use alternative medication		1.7%	0.9%–3.0%
			Not determined		4.9%	3.1%–6.3%
			Use as directed		67.6%	63.6%–70.9%
Opioids	Codeine/Tramadol	East Asian	Avoid and/or use alternative medication	21,185	1.5%	1.4%–1.7%
			Not determined		6.7%	6.3%–7.0%
			Use as directed		53.2%	52.5%–53.9%
			Use with caution		38.5%	37.8%–39.1%
		Japanese	Avoid and/or use alternative medication	632	0.9%	0.7%–2.6%
			Not determined		3.0%	1.9%–4.5%
			Use as directed		71.0%	65.3%–72.5%
			Use with caution		0.9%	23.4%–30.3%
		Kisoi, et al. (2020)	Avoid and/or use alternative medication	216	1.9%	0.7%–4.7%
			Not determined		0.0%	0.6%–0.6%
			Use as directed		74.2%	70.4%–77.5%
			Use with caution		24.1%	21.3%–27.6%
SSRIs	Sertraline	East Asian	Adjust dosage	32,510	52.8%	52.3%–53.4%
			Avoid and/or use alternative medication		15.0%	14.6%–15.4%
			Not determined		0.0%	0.0%–0.0%
			Use as directed		32.2%	31.7%–32.7%
		Japanese	Adjust dosage	632	50.8%	48.0%–55.8%
			Avoid and/or use alternative medication		17.6%	14.3%–20.2%
			Not determined		0.0%	0.0%–0.6%
			Use as directed		31.6%	27.5%–34.7%
	Citalopram/Escitalopram	East Asian	Adjust dosage	32,510	46.0%	45.5%–46.5%
			Avoid and/or use alternative medication		15.6%	15.2%–16.0%
			Not determined		0.3%	0.3%–0.4%
			Use as directed		38.1%	37.5%–38.6%
		Japanese	Adjust dosage	631	46.1%	43.4%–51.1%
			Avoid and/or use alternative medication		17.7%	14.5%–20.4%
			Not determined		0.0%	0.0%–0.6%
			Use as directed		36.1%	31.9%–39.4%

To focus on the most prevalent categories, we restricted analyses within each drug context to the two most frequent recommendation categories for the East Asian and Japanese populations. For each selected category, we quantified the between-population effect size using the risk difference (RD = p_JPN − p_EAS) and computed 95% confidence intervals using the Wald (asymptotic normal) method. We assessed statistical evidence for differences in proportions using a two-proportion z-test and controlled for multiple comparisons across tested categories using the Benjamini–Hochberg (BH) procedure (FDR) (Benjamini and Hochberg, 1995), implemented via statsmodels. stats.multitest.multipletests with method = “fdr_bh” (Seabold and Perktold, 2010). We considered an absolute RD ≥ 5% points as practically meaningful and q < 0.05 as statistically significant. Table 2 summarizes the resulting RD estimates and FDR-adjusted q-values.3.1 SSRIs.

**TABLE 2:** Japanese vs. East Asian CPIC recommendation frequencies for the two most common categories per drug context. RD (p_JPN − p_EAS) and Wald 95% CIs are shown; BH-adjusted q-values were obtained by applying Benjamini–Hochberg FDR correction to two-proportion z-test p-values across all rows.

			Percent		Sample size			
Drug category	Drug	Recommendation	Japanese	East Asian	Japanese	East Asian	RD (95% CI)	BH adjusted q
Opioids	Codeine/Tramadol	Use as directed	71.0%	53.2%	632	21,185	18% (14%–21%)	<0.001
	Codeine/Tramadol	Use with caution	25.0%	38.5%	632	21,185	−13% (−17% to −10%)	<0.001
Statins	Lovastatin/Pitavastatin/Simvastatin	Use as directed	67.6%	75.7%	632	2,471	−8% (−12% to −4%)	<0.001
	Lovastatin/Pitavastatin/Simvastatin	Avoid and/or use alternative medication	27.5%	23.5%	632	2,471	4% (0%–8%)	0.06
	Pravastatin	Use as directed	67.6%	75.7%	632	2,471	−8% (−12% to −4%)	<0.001
	Pravastatin	Use with caution	25.8%	21.9%	632	2,471	4% (0%–8%)	0.06
	Atorvastatin	Use as directed	67.6%	75.7%	632	2,471	−8% (−12% to −4%)	<0.001
	Atorvastatin	Adjust dosage	25.8%	21.9%	632	2,471	4% (0%–8%)	0.06
SSRIs	Sertraline	Use as directed	31.6%	32.2%	632	32,510	−1% (−4% – 3%)	0.85
	Sertraline	Adjust dosage	50.8%	52.8%	632	32,510	−2% (−6% – 2%)	0.39
	Citalopram/Escitalopram	Use as directed	36.1%	38.1%	631	32,510	−2% (−6% – 2%)	0.39
	Citalopram/Escitalopram	Adjust dosage	46.1%	46.0%	631	32,510	0% (−4% – 4%)	0.96

Overall, the Japanese Population dataset is consistent with the CPIC East Asian dataset showing no major divergences, as can be seen in Table 2. Three drugs were considered in the SSRI group, Sertraline, Citalopram and Escitalopram. Results differ between sertraline and Citalopram/Escitalopram as sertraline recommendations are based off of CYP2B6 and CYP2C19 while Citalopram/Escitalopram considers only CYP2C19 (Bousman et al., 2023). This leads to minor changes for the “Adjust Dosage” and “Use as Directed” recommendations for Sertraline and Citalopram/Escitalopram within the same population, for example, a frequency of ∼53% vs. ∼46% respectively in the East Asian population. These results are not show by the analysis to be statistically significant or impactful and therefore these results provide no evidence that the broader East Asian grouping is inaccurate for the Japanese population.

### 3.1 Opioids

Table 1 shows a divergence between CPIC’s East Asian population and A. D.A.M. Innovations’ Japanese population with supporting data from a third source for the two opioid drugs, codeine and tramadol. For these opioids, CYP2D6 phenotype is used to determine the PGx classification (Crews et al., 2021). The Japanese population saw an increase in the “Use as directed” designation compared to the East Asian population from 53.2% to 71.0% and a concomitant decrease in the “Use with Caution” and “Not Determined” designations. Table 2 shows that this is the BH-adjusted q-values indicate FDR significant divergence, with q-values below 0.001. For the CPIC East Asian dataset, the population labeled “Japanese” was 5,500 individuals out of a total of 21,185 (25.8%) (Gene Reference Materials for CYP2D6, 2022). This would dilute any uniquely Japanese signal and lead to the observed divergence. To validate these differences, the CPIC diplotype frequencies were compared to a third dataset directly measuring the CYP2D6 genotype. Kisoi et al. genotyped 216 Japanese women covering the *1, *2, *5, *10, *14 and *41 genotypes (Kisoi et al., 2020). The diplotypes provided were converted into activity scores and PGx recommendations to allow for comparison to the existing data. This followed the same approach as harmonizing the Japanese dataset with the CPIC recommendations we used the tables for CYP2D6 (Gene Reference Materials for CYP2D6, 2022) and the publication for Opioids (Crews et al., 2021). From Table 1, we can see frequencies for “Use as Directed” of 71.0% and 74.2% for the A. D.A.M. Innovations and Kisoi et al. data, with overlap in the confidence interval. There is consistency between the A. D.A.M. Innovations data and Kisoi’s data, indicating that there is a divergence between the Japanese population and the East Asian population. This conclusion should be tempered by the female only sample, small sample size and limited genotypes in the Kisoi paper. The conversion of the Kisoi et al. data is included in Table 1. These results align with previous data and provide a better understanding of the Japanese populations PGx relevant genes.

### 3.2 Statins

The SLCO1B1 phenotype determines the PGx classification for the statins considered here. However, there is variation in what classification is associated with which phenotype, leading to consistent “Use as Directed” recommendations, but shifts between “Avoid and/or Use Alternative Medication,” “Use with Caution” and “Adjust Dosage” recommendations as shown in Figure 1 (Cooper-DeHoff et al., 2022). The statins’ data suggests a difference between the East Asian and Japanese populations, as shown in Table 1. The frequency of “Use as Directed” is higher in the East Asian population (75.7%) vs. all statins considered (67.6%). The non-overlapping confidence intervals suggest a meaningful difference. Table 2 shows that further analysis confirms this conclusion. Table 2 however does not confirm that the “Adjust dosage”, “Use With Caution,” and “|Avoid and/or Use Alternative Medication” recommendations for atorvastatin, pravastatin and Lovastatin/pitavastatin/simvastatin respectively; these will need further research to confirm the difference. The BH-adjusted q-values fail to meet the requirements for significance (q < 0.005) and the RD value is above our cutoff for a practically meaningful difference. However, an independent source of data could not be found to corroborate this difference, indicating an avenue for future research and confirmation.

These comparative analyses demonstrate the scientific value of Japanese-specific PGx data and contextualize global guidelines. Additionally, they open future possibilities for refining drug labels, clinical decision support, and pharmacovigilance in Japan.

## 4 Discussion

Understanding population variation in PGx relevant phenotype frequencies can have clinical and research relevance (Hiratsuka et al., 2021; Li et al., 2023). CPIC provides this data through published guidelines for 35 genes and 165 drugs, many of which include geographic population data (Relling et al., 2020). As an example, CPIC has phenotype frequencies for CYP2D6 across multiple populations (Gene Reference Materials for CYP2D6, n. d). The “American” category includes Inuit populations in Canada (Jurima-Romet et al., 1997) to various native American populations in Argentina and Paraguay (Bailliet et al., 2007). Such a grouping may show a variation between “American” and “European” populations but obscures the probable variations between populations spanning two separate continents. Being able to further parse these large populations into meaningful sub-populations would be useful in itself.

The Japanese population’s demographic history allows a unique opportunity to show within CPIC population variation. The demographic history is often described with the dual-structure model, which posits that the modern Japanese population is an admixture of Jomon and Yayoi, earlier and later inhabitants of the Japanese archipelago, respectively (Hanihara, 1994; Hudson et al., 2020). Yet the published pharmacogenomic resources, such as CPIC, often collapse Japanese data into broader East Asian aggregates, blurring signals that are crucial for precise clinical recommendations. Because much effort has been placed in clarifying the genetic history of the population of the Japanese Archipelago, we can expect this history to lead to founder effects or historical bottle necks that influence the PGx relevant genes such as CYP2D6, CYP2C19 and SLCO1B1. With the broad East Asian grouping that CPIC uses where the Japanese percentage varies from ∼20% to ∼35% of the total, this blurring occurs (Gene Reference Materials for CYP2D6, 2022; Gene Reference Materials for CYP2C19, 2020; Gene Reference Materials for SLCO1B1, 2024). As PGx advances, there are efforts to create finer-grained groups. As an example, the Japanese Pharmacogenetics Clinical Implementation Consortium (JCPIC) is working to make accessible and localize the available PGx recommendations for the Japanese population (JCPIC Activities, 2024). For population like the Japanese with a unique genetic history this could lead to unforeseen and undesirable pharmacological effects.

The approach presented here extracts additional conclusions for the Japanese population from the existing dataset, therefore we must differentiate between two types of pipelines: A primary pipeline, that delivers an analysis of an individual’s PGx results and a secondary pipeline that delivers population level data using aggregated data from the primary pipeline. This should not be conflated with the primary, secondary and tertiary analysis portions of a bioinformatics pipeline (Austin-Tse et al., 2022). The primary and secondary pipelines in this paper should be considered part of the tertiary analysis where the data relevant to the individual and secondary findings relevant to populations are collated. The value of this distinction is in how it makes clear that additional data is being extracted or reframed from the original datasets. It breaks down a silo between individuals’ data to allow for a wide range of additional conclusions to be reached. However, considering the removal of data siloing care must be taken to anonymize and protect user privacy.

To ensure privacy, any connection between kit ids and individuals’ details that connect to the wider database and the personal information stored there were removed. In addition, time stamps were dropped from tables containing customer data to prevent them from acting as a proxy for ids. The guiding principle is to preserve privacy while enabling maximum scientific value from dormant data. We want to avoid non-authorized users from re-identification of individuals via linkage in the case of a leak of user data. By making only this data available we wish to minimize the damage of any possible future data leak. Limiting the release of the data to population level in this paper and only releasing the privacy protecting data set to those who request it makes it possible to meet the scientific demands of the community while respecting the privacy of the participants. Only after addressing these concerns can the database be shared and analyzed.

The final dataset of this secondary pipeline opens several avenues for research and direct application. Clinical guidelines could be refined. Japanese-specific data can inform updates to CPIC and other international PGx guidelines, ensuring they reflect real-world allele frequencies rather than pan-Asian assumptions. In turn, the data could refine the drug discovery process. Pharmaceutical companies can use population-specific PGx data to identify risk alleles early in development, anticipate adverse events, and design safer clinical trials. Finally, it can provide additional information for precision public health decisions. Aggregated data on drug response across age, sex, and population can guide health policy, from screening recommendations to formulary design. Most importantly, this work demonstrates that secondary pipelines transform dormant genomic data into functional insights.

**FIGURE 2:**
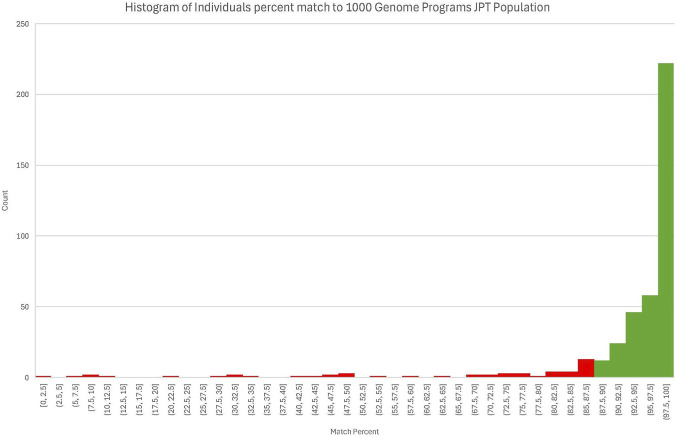
Histogram of all individuals before filtering by Japanese genetic ancestry based on percent match to the 1,000 Genome Project’s Japanese in Tokyo, Japan data set. Retained individuals are highlighted in green and have a match percentage above 87.5.

However, it is important to explicitly note the limitations of this initial study. The sample size is relatively small (only 632 individuals) and is not randomly selected. The members of the initial population have all selected to purchase a whole genome sequence consumer testing service. This could introduce bias into the dataset by selecting for socioeconomic status or some other unforeseen variable. Another possible source of bias is the reference database used to select the sample set. Any biases in that dataset would propagate through the project. The full A. D.A.M. Innovations WGS dataset was filtered by estimated Japanese genetic ancestry based on 1000 Genomes Project data (‘Japanese in Tokyo, Japan [JPT]’), requiring an 87.5% match or greater. This criterion approximates the 1000 Genomes Project’s requirement of having all four grandparents born in Japan, while accommodating minor levels of admixture, such as one great grandparent born elsewhere. Figure 2 shows that the plurality of the data clusters near 100 and tales off towards lower values. Changing the threshold by +/-2.5% would only change the count by less than 15 individuals. The selection of 87.5% as the cut off is well supported conceptually from the 1,000 Genome Projects approach and by consideration of the distribution of the data displayed in Figure 2. Although Tokyo has seen net internal migration from other regions in Japan, it is unlikely that this net migration has perfectly replicated the variation across the archipelago (Fielding, 2024; Kakinuma and Abel, 2022). The result is a dataset that prioritizes genetic homogeneity for population inference while maintaining inclusivity. Also, the 1000 Genomes Project sets a practical limit to the strictness of our filter, as it had to make its own determination on how to classify an individual as Japanese. By setting genetic ancestry thresholds at the start, the pipeline ensures that subsequent PGx allele frequencies and phenotype distributions reflect the Japanese population specifically, rather than diluted pan-Asian aggregates. This makes the database uniquely valuable for both local clinical practice and international comparisons.

Another limitation to consider is that this approach reorders existing data to derive new conclusions, inherently it cannot analyze data that is not present in the initial dataset. Consider two cases that maybe useful for population level analyses: Mosaic Loss of Y (MLOY) (Forsberg et al., 2014) and Principal Component analysis (PCA) for inferred ancestry (Price et al., 2010). MLOY is the loss of the Y chromosome in some leukocytes over time and has been connected with negative health outcomes. This information could be calculated from the raw WGS data that the initial dataset is constituted from. However, if that data was not included in the initial data set a secondary pipeline cannot derive it. A new primary pipeline would have to go back to the original raw files and extract the MLOY measurements. Likewise, a PCA analysis of the populations discussed in this paper, particularly the inferred Japanese Population from the secondary pipeline, could bring additional confirmation of the approach. However, that would require going beyond the primary dataset to the raw files and would constitute a primary pipeline. This is a fundamental limitation of the secondary pipeline approach.

In conclusion, the pipeline demonstrates four relevant results. First, it establishes a Japanese-specific pharmacogenomic database filtered for genetic ancestry thresholds, creating a unique and underrepresented resource. Second, it describes a reproducible, privacy-conscious secondary pipeline that transforms individual WGS results into aggregated, query-ready SQLite databases. Third, it demonstrates proof-of-concept comparisons between Japanese data and CPIC populations across SSRIs, statins, and opioids, illustrating where Japanese frequencies diverge from East Asian aggregates. Finally, it has broader significance, emphasizing the clinical and scientific opportunities unlocked by secondary pipelines, including refining global PGx guidelines, enhancing drug development, and expanding conversations about gene–drug interactions. By leading with Japanese genetic ancestry and building toward pipeline design and comparative analysis, this study illustrates both the technical feasibility and scientific necessity of secondary pipelines. It demonstrates a model for how localized, ancestry or population-specific pharmacogenomic databases can contribute to functional insights, bridging the gap between individual patient reports and population-scale trends.

## Data Availability

The raw data supporting the conclusions of this article will be made available by the authors, without undue reservation.
