# Supplementary material for: Japanese genomes for pharmacogenomics: primary and secondary pipelines for population-specific insights
Source: Front Bioinform. 2026 Mar 19;6:1770550. doi: 10.3389/fbinf.2026.1770550 (PMC13044107; doi:10.3389/fbinf.2026.1770550)
Supplement: Supplementary file 1 [file Supplementaryfile1.docx]

Supplementary Material

# Supplementary Figures

## Database Structure


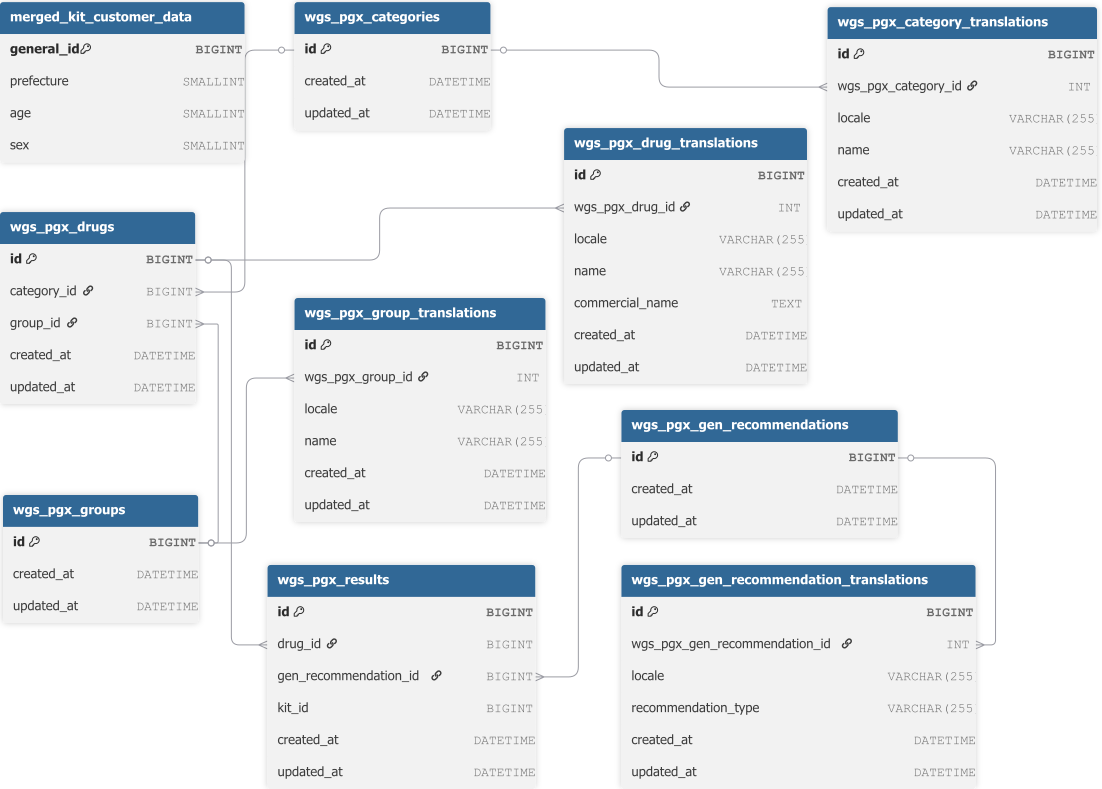


**Supplementary Figure 1**

## Metabase example


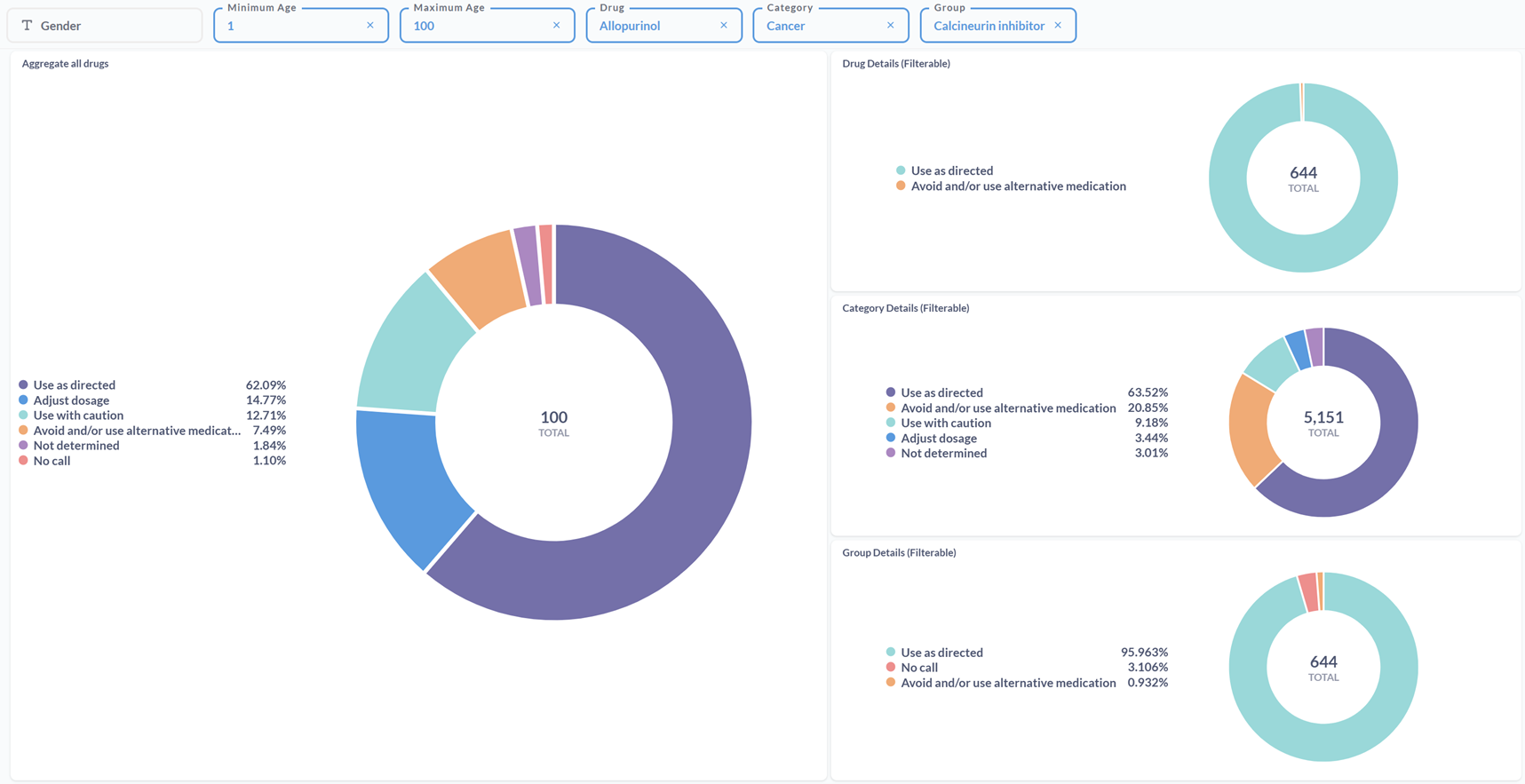


**Supplementary Figure 2.**

# Supplementary Tables

## SSRI Frequencies

| Drug | Population | Phenotype | Frequency |
| --- | --- | --- | --- |
| Sertraline | Central/South Asian | Adjust dosage | 46.0% |
|  |  | Avoid and/or use alternative medication | 12.9% |
|  |  | Not Determined | 0.0% |
|  |  | Use as directed | 41.2% |
|  | East Asian | Adjust dosage | 52.8% |
|  |  | Avoid and/or use alternative medication | 15.0% |
|  |  | Not Determined | 0.0% |
|  |  | Use as directed | 32.2% |
|  | Japanese | Adjust dosage | 51.9% |
|  |  | Avoid and/or use alternative medication | 17.0% |
|  |  | Not Determined | 0.0% |
|  |  | Use as directed | 31.0% |
|  | European | Adjust dosage | 39.8% |
|  |  | Avoid and/or use alternative medication | 7.1% |
|  |  | Not Determined | 0.0% |
|  |  | Use as directed | 53.1% |
|  | Sub-Saharan African | Adjust dosage | 47.8% |
|  |  | Avoid and/or use alternative medication | 13.5% |
|  |  | Not Determined | 0.0% |
|  |  | Use as directed | 38.7% |
| Citalopram/ Escitalopram | Central/South Asian | Adjust dosage | 40.8% |
|  |  | Avoid and/or use alternative medication | 29.6% |
|  |  | Not determined | 0.0% |
|  |  | Use as directed | 29.6% |
|  | East Asian | Adjust dosage | 46.0% |
|  |  | Avoid and/or use alternative medication | 15.6% |
|  |  | Not determined | 0.3% |
|  |  | Use as directed | 38.1% |
|  | Japanese | Adjust dosage | 47.2% |
|  |  | Avoid and/or use alternative medication | 17.2% |
|  |  | Not determined | 0.0% |
|  |  | Use as directed | 35.6% |
|  | European | Adjust dosage | 26.2% |
|  |  | Avoid and/or use alternative medication | 34.2% |
|  |  | Not determined | 0.0% |
|  |  | Use as directed | 39.6% |
|  | Sub-Saharan African | Adjust dosage | 34.2% |
|  |  | Avoid and/or use alternative medication | 28.8% |
|  |  | Not determined | 0.0% |
|  |  | Use as directed | 37.0% |

**Supplementary Table 1.**

## Statins Frequencies

| Drug | Population | Phenotype | Frequency |
| --- | --- | --- | --- |
| lovastatin/  pitavastatin/ simvastatin | Central/South Asian | Avoid and/or use alternative medication | 13.5% |
|  |  | Not determined | 0.1% |
|  |  | Use as directed | 86.5% |
|  | East Asian | Avoid and/or use alternative medication | 23.5% |
|  |  | Not determined | 0.8% |
|  |  | Use as directed | 75.7% |
|  | Japanese | Avoid and/or use alternative medication | 28.3% |
|  |  | Not determined | 4.4% |
|  |  | Use as directed | 67.3% |
|  | European | Avoid and/or use alternative medication | 31.2% |
|  |  | Not determined | 0.1% |
|  |  | Use as directed | 68.6% |
|  | Sub-Saharan African | Avoid and/or use alternative medication | 5.5% |
|  |  | Not determined | 0.0% |
|  |  | Use as directed | 94.5% |
| pravastatin | Central/South Asian | Avoid and/or use alternative medication | 0.5% |
|  |  | Not determined | 0.1% |
|  |  | Use as directed | 86.5% |
|  |  | Use with caution | 13.0% |
|  | Japanese | Avoid and/or use alternative medication | 1.7% |
|  |  | Not determined | 4.4% |
|  |  | Use as directed | 67.3% |
|  |  | Use with caution | 26.6% |
|  | East Asian | Avoid and/or use alternative medication | 1.6% |
|  |  | Not determined | 0.8% |
|  |  | Use as directed | 75.7% |
|  |  | Use with caution | 22.0% |
|  | European | Avoid and/or use alternative medication | 2.9% |
|  |  | Not determined | 0.1% |
|  |  | Use as directed | 68.6% |
|  |  | Use with caution | 28.3% |
|  | Sub-Saharan African | Avoid and/or use alternative medication | 0.1% |
|  |  | Not determined | 0.0% |
|  |  | Use as directed | 94.5% |
|  |  | Use with caution | 5.4% |
| atorvastatin | Central/South Asian | Adjust dosage | 13.0% |
|  |  | Avoid and/or use alternative medication | 0.5% |
|  |  | Not determined | 0.1% |
|  |  | Use as directed | 86.5% |
|  | East Asian | Adjust dosage | 22.0% |
|  |  | Avoid and/or use alternative medication | 1.6% |
|  |  | Not determined | 0.8% |
|  |  | Use as directed | 75.7% |
|  | Japanese | Adjust dosage | 26.6% |
|  |  | Avoid and/or use alternative medication | 1.7% |
|  |  | Not determined | 4.4% |
|  |  | Use as directed | 67.3% |
|  | European | Adjust dosage | 28.3% |
|  |  | Avoid and/or use alternative medication | 2.9% |
|  |  | Not determined | 0.1% |
|  |  | Use as directed | 68.6% |
|  | Sub-Saharan African | Adjust dosage | 5.4% |
|  |  | Avoid and/or use alternative medication | 0.1% |
|  |  | Not determined | 0.0% |
|  |  | Use as directed | 94.5% |

**Supplementary Table 2.**

## Opioids Frequencies

| Drug | Population | Phenotype | Frequency |
| --- | --- | --- | --- |
| Codeine/ Tramadol | Central/South Asian | Avoid and/or use alternative medication | 3.9% |
|  |  | Not determined | 10.0% |
|  |  | Use as directed | 58.1% |
|  |  | Use with caution | 28.1% |
|  | East Asian | Avoid and/or use alternative medication | 1.5% |
|  |  | Not determined | 6.7% |
|  |  | Use as directed | 53.2% |
|  |  | Use with caution | 38.5% |
|  | Japanese | Avoid and/or use alternative medication | 1.4% |
|  |  | Not determined | 2.9% |
|  |  | Use as directed | 69.0% |
|  |  | Use with caution | 26.7% |
|  | European | Avoid and/or use alternative medication | 8.7% |
|  |  | Not determined | 3.7% |
|  |  | Use as directed | 49.2% |
|  |  | Use with caution | 38.3% |
|  | Sub-Saharan African | Avoid and/or use alternative medication | 6.0% |
|  |  | Not determined | 9.0% |
|  |  | Use as directed | 46.4% |
|  |  | Use with caution | 38.1% |

**Supplementary Table 3.**
